# NMR-Metabolomics Reveals a Metabolic Shift after Surgical Resection of Non-Small Cell Lung Cancer

**DOI:** 10.3390/cancers15072127

**Published:** 2023-04-03

**Authors:** Elien Derveaux, Melvin Geubbelmans, Maarten Criel, Ingel Demedts, Ulrike Himpe, Kurt Tournoy, Piet Vercauter, Erik Johansson, Dirk Valkenborg, Karolien Vanhove, Liesbet Mesotten, Peter Adriaensens, Michiel Thomeer

**Affiliations:** 1 Faculty of Medicine and Life Sciences, Hasselt University, Martelarenlaan 42, B-3500 Hasselt, Belgium; elien.derveaux@uhasselt.be (E.D.);; 2 Applied and Analytical Chemistry, Institute for Materials Research, Hasselt University, Agoralaan 1—Building D, B-3590 Diepenbeek, Belgium; 3 Data Science Institute, Hasselt University, Agoralaan 1, B-3590 Diepenbeek, Belgium; 4 Interuniversity Institute for Biostatistics and Statistical Bioinformatics (I-BioStat), Agoralaan 1, B-3590 Diepenbeek, Belgium; 5 Department of Respiratory Medicine, Ziekenhuis Oost-Limburg, Schiepse Bos 6, B-3600 Genk, Belgium; 6 Department of Respiratory Medicine, AZ Delta, Deltalaan 1, B-8800 Roeselare, Belgium; 7 Department of Respiratory Medicine, Onze-Lieve-Vrouw Ziekenhuis, Moorselbaan 164, B-9300 Aalst, Belgium; 8 Faculty of Medicine and Health Sciences, Ghent University, De Pintelaan 85, B-9000 Ghent, Belgium; 9 Sartorius Stedim Data Analytics AB, Östra Strandgatan 24, 903 33 Umeå, Sweden; 10 Department of Respiratory Medicine, AZ Vesalius, Hazelereik 51, B-3700 Tongeren, Belgium; 11 Department of Nuclear Medicine, Ziekenhuis Oost-Limburg, Schiepse Bos 6, B-3600 Genk, Belgium

**Keywords:** non-small cell lung cancer, NMR spectroscopy, metabolomics

## Abstract

**Simple Summary:**

The initiation of non-small-cell lung cancer (NSCLC) causes significant changes in a patient’s blood plasma metabolomic profile. Locally and early-advanced NSCLC patients receive a complete surgical resection of the lung tumor, but the level of metabolic changes after this surgical treatment is currently unknown. By collecting multiple blood plasma samples before and after complete NSCLC removal, metabolic changes can be detected by analyzing the patient’s plasma using proton nuclear magnetic resonance (NMR) spectroscopy. Detection of significant changes in the plasma metabolism, a so-called metabolic shift in the patient’s blood plasma after surgical tumor resection, indicates the absence of disease recurrence and thus can provide an indication of a good prognosis.

**Abstract:**

Background: Lung cancer can be detected by measuring the patient’s plasma metabolomic profile using nuclear magnetic resonance (NMR) spectroscopy. This NMR-based plasma metabolomic profile is patient-specific and represents a snapshot of the patient’s metabolite concentrations. The onset of non-small cell lung cancer (NSCLC) causes a change in the metabolite profile. However, the level of metabolic changes after complete NSCLC removal is currently unknown. Patients and methods: Fasted pre- and postoperative plasma samples of 74 patients diagnosed with resectable stage I-IIIA NSCLC were analyzed using ^1^H-NMR spectroscopy. NMR spectra (*s* = 222) representing two preoperative and one postoperative plasma metabolite profile at three months after surgical resection were obtained for all patients. In total, 228 predictors, i.e., 228 variables representing plasma metabolite concentrations, were extracted from each NMR spectrum. Two types of supervised multivariate discriminant analyses were used to train classifiers presenting a strong differentiation between the pre- and postoperative plasma metabolite profiles. The validation of these trained classification models was obtained by using an independent dataset. Results: A trained multivariate discriminant classification model shows a strong differentiation between the pre- and postoperative NSCLC profiles with a specificity of 96% (95% CI [86–100]) and a sensitivity of 92% (95% CI [81–98]). Validation of this model results in an excellent predictive accuracy of 90% (95% CI [77–97]) and an AUC value of 0.97 (95% CI [0.93–1]). The validation of a second trained model using an additional preoperative control sample dataset confirms the separation of the pre- and postoperative profiles with a predictive accuracy of 93% (95% CI [82–99]) and an AUC value of 0.97 (95% CI [0.93–1]). Metabolite analysis reveals significantly increased lactate, cysteine, asparagine and decreased acetate levels in the postoperative plasma metabolite profile. Conclusions: The results of this paper demonstrate that surgical removal of NSCLC generates a detectable metabolic shift in blood plasma. The observed metabolic shift indicates that the NSCLC metabolite profile is determined by the tumor’s presence rather than donor-specific features. Furthermore, the ability to detect the metabolic difference before and after surgical tumor resection strongly supports the prospect that NMR-generated metabolite profiles via blood samples advance towards early detection of NSCLC recurrence.

## 1. Introduction

Metabolic reprogramming is a key hallmark of cancer initiation, progression, and relapse [1,2,3]. Nutrient-deprived conditions and hypoxia initiate the loss of tumor suppressor genes and activation of oncogenes to sustain the high proliferation rates observed in lung cancer cells, promoting metabolic reprogramming within a common set of fundamental metabolic pathways [4]. However, the metabolism of individual lung cancer patients is defined explicitly by the heterogeneity of these pathways’ metabolic flux dynamics and alterations in nutrient use [5,6]. Nuclear magnetic resonance (NMR)-metabolomics analyses of biofluids, such as plasma, allow the determination of a precise metabolic snapshot representing the current metabolic state of an individual, with or without a disease [7,8]. This NMR-obtained plasma metabolite profile or fingerprint typically represents the individual’s plasma metabolite concentrations and hereby provides an insight into possible changes in biochemical reactions [9,10].

The metabolite profile is reported to have a donor-intrinsic character, as confirmed by Assfalg et al. They demonstrated the existence of such a donor-specific urine metabolite fingerprint with a 100% identification probability [11]. Furthermore, these donor-specific metabolite profiles are generally stable over a long-term period, as repeatedly demonstrated from samples taken two days to ten years apart [11,12,13,14,15,16,17,18,19,20]. Influences by daily variations caused by environmental factors such as nutritional status seem to be accounted for by the flexibility (i.e., metabolic resilience) within the individual limits of the metabolite profile [21,22]. However, the onset of a pathological disease that causes larger variations will force the donor-specific metabolite profile out of its individual metabolic range [18,19]. Moreover, after monitoring 12 individuals for ten years, it was suggested by Ghini et al. that the individual metabolite profile could be restored when the pathological condition is over [18]. This indicates that the personal metabolite profile can be used to monitor, follow-up, and maybe even predict the patient’s treatment response which can improve patient stratification during treatment follow-up [23,24]. For example, by analyzing circulating tumor DNA (ctDNA) via liquid biopsies, the correct selection of patients who would benefit from targeting minimal residual disease (MRD) by adjuvant chemotherapy could lower the disease recurrence rate and improve non-small-cell lung cancer (NSCLC) outcome [25,26,27]. Thus, the detection of significant changes in the personal metabolite profile might also be critical for post-surgical treatment decisions and monitoring locally and early-advanced stage NSCLC patients, for whom the standard-of-care therapy is complete surgical resection. Once the surgery is performed, the individual metabolite profile can be monitored in real-time and, as such, might detect early disease recurrence in a minimally invasive manner.

This study aims to determine the effect of complete surgical removal on the plasma metabolite profile of locally and early-advanced-stage NSCLC patients without rapid NSCLC recurrence. By prospectively collecting blood samples at specific pre- and postoperative time points from all eligible patients, patient-specific plasma metabolite profiles before and after surgery can be compared. While it has been reported that the metabolite profile is patient-specific and changes in the function of an acute stress response, the level of metabolic changes after surgical resection of lung cancer, remains currently unknown.

## 2. Materials and Methods

### 2.1. Ethical Statement

Samples were collected during the registered trial with the study number NCT03736993, conducted following the Helsinki Declaration and Good Clinical Practice’s ethical rules. The study protocol was approved by the Medical Committees of ZOL (Ziekenhuis Oost-Limburg, Genk, Belgium), AZDelta (Roeselare, Belgium), OLV Ziekenhuis (Onze-Lieve-Vrouw Ziekenhuis, Aalst, Belgium) and Hasselt University (Hasselt, Belgium). Signed informed consent was obtained from all participants before inclusion.

### 2.2. Study Participants

**Figure 1:**
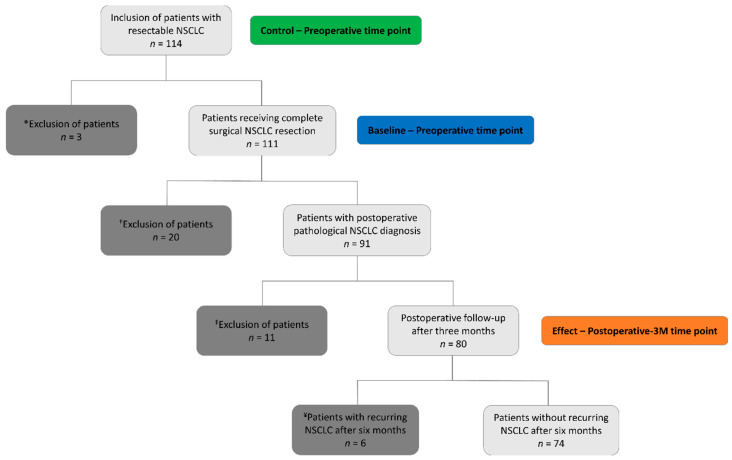
Study flow diagram showing the overview of patient recruitment at Ziekenhuis Oost-Limburg (ZOL) from June 2018 until August 2021 of the NCT03736993 trial with the indication of the three blood sampling time points (control, baseline and effect). The inclusion stop between March and June 2020 due to the COVID-19 pandemic had no further impact on the study participants’ planned blood sampling time points. * Surgery was cancelled or excluded due to patient drop-out. † Postoperative pathological diagnosis of a non-malignant lesion or another type of cancer. *, † Patients were excluded from the NCT03736993 trial and no further blood sampling occurred. ‡ Datasets from at least one of the three time points were missing due to high glycemic values (>200 mg/dL) or exclusion due to patient drop-out. ‡, ¥ Patients were excluded from this explorative sub-study, but not from the NCT03736993 clinical trial.

All study participants were diagnosed with resectable stage I-IIIA NSCLC. The inclusion criteria were (i) no history of, or treatment for, cancer during the last five years, (ii) fasted and no medication intake for at least six hours and (iii) a fasting glucose concentration below 200 mg/dL. Patients who did not receive complete surgical resection or with a postoperative pathological non-malignant diagnosis were excluded from the study. Specifically for this sub-study, all study participants without cancer recurrence six months after surgical resection were selected. The sample size of this sub-study is based on the feasibility and availability of the study participants. An inclusion stop between March and June 2020 due to the COVID-19 pandemic had no further impact on the participants’ planned blood sampling time points. The complete sub-study flow diagram is shown in Figure 1. Demographics and clinical characteristics of the 74 eligible study participants in the training (*n* = 50) and validation (*n* = 24) cohort are summarized in Table 1.

### 2.3. Pre-Analytical Plasma Sample Preparation

All venous blood samples were collected in 6 mL spray-dried lithium-heparin tubes and stored immediately on ice to delay metabolic activity [28]. Within 4 h after collection, cooled blood samples were centrifuged for 15 min at 1600× *g*, and plasma aliquots of 400 µL were stored at −80 °C until the proton ^1^H-NMR analysis.

### 2.4. ^1^H-NMR Analysis and Processing

All plasma samples were further prepared and analyzed using ^1^H-NMR spectroscopy as described in the protocol of Derveaux et al. [29]. In short, plasma samples were thawed and 350 µL plasma was added to 350 µL 0.15 M potassium phosphate buffer pH 7.4 in D_2_O containing 8 mM trimethylsilyl-2,2,3,3-tetradeuteropropionic acid (TSP) and 62.50 µM maleic acid (MA) as an internal standard for quantification. All ^1^H-NMR spectra were acquired on a 600 MHz JEOL NMR spectrometer using a 5 mm Royal HFX probe. A CPMG pulse sequence with a total spin-echo time of 64 ms, a spin-echo delay of 0.4 ms, and 160 loops was used to attenuate signals of remaining plasma proteins with very short T_2_ relaxation times. Other parameters were: presaturation for 3 s to accomplish water suppression and an acquisition time of 2.27 s using 16 k data points. Afterwards, all spectra were processed using JEOL Delta Software (version 5.3.1) using a line-broadening value of 0.8 Hz and a zero-filling factor of 4 to acquire 64 k data points. All spectra were Fourier transformed, phased manually and baseline corrected. Chemical shift ppm calibration was done using the upfield peak of the methyl doublet of the alanine signal which was set at 1.4938 ppm. Since most metabolites contain hydrogen in several chemical environments, they will also give rise to more than one signal. In the used methodology, the spectra were divided into 237 well-defined regions, representing the proton signals of 62 metabolites. Each region was then integrated (area under the peaks in the defined region), and these integration values were normalized to the integration value of maleic acid (internal standard for quantification; added in a known concentration of 31.25 µg/mL). This way, 237 variables (i.e., integration values), or predictors are created that represent the metabolite concentrations used for the multivariate statistical analysis. Nine of these variables were excluded from the analysis due to a %RSD > 10%, as explained in the protocol [29]. The resulting 228 variables together represent the patient’s plasma metabolite profile at a specific time point. For all 74 NSCLC patients, a plasma metabolite profile is available at the baseline (B; day of surgery; preoperative), control (C; after the diagnosis but before surgery; preoperative) and effect (E, three months after surgery; postoperative-3M) sampling time point, providing 222 complete metabolic datasets, each with 228 variables, for further multivariate statistical analysis.

### 2.5. Data Analysis by Multivariate and Univariate Statistics

The stepwise statistical analysis consists of four main steps: (i) multivariate discriminant statistics using supervised OPLS-DA and OPLS-EP analyses, (ii) identification of most differentiating variables using VIP analysis, (iii) further selection of differentiating variables that are highly selective for a single metabolite. and corresponding metabolite assignment based on the method-specific database developed by metabolite spiking experiments and (iv) testing pre- and postoperative metabolite concentration differences by univariate statistics for paired samples.

#### 2.5.1. Multivariate Discriminant Analysis

Supervised discriminant analysis such as OPLS-DA (orthogonal partial least squares discriminant analysis) is a common strategy applied in NMR-metabolomics, where a high number of spectral intensity values are often used to find a discriminating driving force between the two groups [30]. The analysis conforms to the reporting standard of the TRIPOD statement [31]. First, a training classification model is constructed using group information (i.e., in this paper: preoperative metabolite profiles during the presence of cancer versus postoperative metabolite profiles without cancer recurrence) to find maximal separation. Then, different model parameters can be used to estimate the quality of the trained classifier: R2X: the goodness of fit (i.e., variation within the groups explained by the model), R2Y: explained variation between the groups, and Q2: predictive accuracy of the trained model. A Q2 value > 0.30 commonly indicates a high predictive ability. Afterwards, this trained classification model can be validated using an independent dataset without providing group information. A validation procedure showing high specificity and sensitivity levels confirms the actual differentiation between the two groups. To visualize multivariate OPLS-DA analyses, a score plot is used to summarize the relationship among the different metabolite profiles. Each metabolite profile is represented by a specific position in the score plot determined by the orthogonal (variation within the groups, vertical axis) and predictive axis (variation between the groups, horizontal axis). The lack of overlap on the predictive axis indicates differentiation between the two groups.

All multivariate discriminant statistical analyses were performed in SIMCA^®^ (version 17.0.1, Sartorius Data Analytics AB, Umeå, Sweden) using the 228 robust variables of the plasma metabolite profile [29]. All variables were unit-variance (UV)-scaled and centered. Seven-fold internal cross-validation was performed to construct the supervised multivariate models. Of the 74 NSCLC patients, 50 patients were randomly selected to train three OPLS-DA models. B/E (*s* = 100; 50 patients at B and 50 patients at E) and C/E (*s* = 100) OPLS-DA classification models were constructed to compare baseline versus effect and control versus effect, respectively. A third B/C (*s* = 100) classifier was trained as an additional control. These trained models are validated using an independent patient cohort consisting of the remaining 24 patients for B/E (*s* = 48) and 23 patients for C/E (*s* = 46; one control sample was identified as an outlier, as shown in Figure S1, and excluded) to confirm the differentiation between the pre- and postoperative time points using the trained OPLS-DA models. Similarly, the two preoperative datasets from the same validation cohort were used to validate the trained B/C model (*s* = 46) as an additional control. A permutation test (500 permutations) of the trained B/E model was used to support the findings of the differentiating models (Figure S2). The negative Q2 value and R2X value > 0.20 of the permutation plots support a strong classification model. Furthermore, a principal component analysis (PCA) model colored for (i) overall pathological tumor stage and (ii) tumor histology was constructed to indicate the absence of confounding effects (Figure S3). Since the metabolic datasets of the same patient cohort are compared pre- and postoperative in each classification model, the samples are paired and dependent. In such cases, OPLS-effect projections (OPLS-EP) models allow the multivariate analysis of paired samples over time [32]. An effect matrix in which the preoperative baseline or control values are subtracted from the matching postoperative-3M values and a y-vector with an identical value for each patient are visualized into an OPLS-EP regression plot. The same 228 variables from 74 patients were UV-scaled without centering on constructing the B/E and C/E OPLS-EP models. The resulting *p*-values from Fisher’s combined probability and CV-ANOVA tests are obtained to confirm the significance of each OPLS-DA and OPLS-EP model, respectively [33]. Receiving operating curves (ROC) were constructed for all trained- and validation OPLS-DA models using R (version 4.1.3). All 228 NMR-generated variables were used to construct cumulative ROC curves using internal validation via non-parametric bootstrapping resampling.

#### 2.5.2. Identification of Differentiating Variables

Variable importance for projection (VIP) analysis was used to identify the 30 variables that contribute the most to the OPLS-DA (Table S1) and OPLS-EP (Table S2) B/E classification models. All 30 variables show a VIP-value > 1. S-plots were used to detect whether the postoperative levels of the identified variables were in- or decreased.

#### 2.5.3. Selection of Differentiating Variables Corresponding to a Single Metabolite

The metabolites corresponding to the proton signals of these 30 integration regions (and so to the corresponding variables or integration values) were assigned based on spiking experiments with 62 known metabolites [29]. Since several of these 30 integration regions contain proton signals from more than one metabolite, spectra of B and E time points were superimposed to determine which signals were increased and decreased. Combining this information with the observed J-coupling patterns, eleven integration regions could be identified that are highly selective for a single metabolite (i.e., integration regions that have no significant contribution from other signals). The above can be done very accurately because the experimental methodology (see Section 2.4) ensures fixed chemical shift (ppm) positions for the metabolite signals without sample-to-sample variation. In this way, four metabolites were identified that differentiate most strongly between the groups: lactate, cysteine, asparagine and acetate. The eleven integration regions (and corresponding variables) are: VAR 039, 040, 041 (lactate, quadruplet signal of ^α^CH); VAR 139, 140, 142, 143 (cysteine, double doublet signal of ^β^CH_2_); VAR 148, 150, 151 (asparagine, double doublet signal of ^β^CH_2_); VAR 199 (acetate, singlet signal of CH_3_) (Table S3).

#### 2.5.4. Univariate Statistics

Normality was tested for these eleven variables using a Kolmogorov–Smirnov test (Table S3). Since all variables show a normal distribution, parametric paired t-tests were used to compare the pre- and postoperative integration values (variables) of the proton signals representing lactate, cysteine, asparagine and acetate. All the integration values were normalized to the integration value of the maleic acid internal standard. For lactate, cysteine, and asparagine, the sum of the integration values of the selected metabolite-specific proton signals was used for univariate comparison.

## 3. Results

### 3.1. Patient Population and Study Design

**Figure 2:**
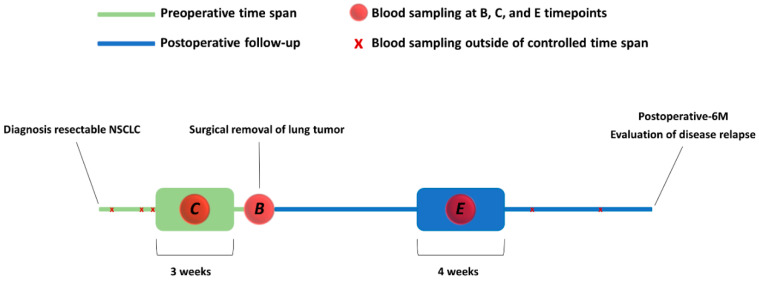
Overview of the longitudinal study design and three different blood sampling time points. In total, three blood samples were donated by 74 NSCLC patients, who were enrolled in a follow-up period of at least six months after surgery. Clinical evaluation of disease relapse was performed six months after surgery. The preoperative blood sampling at baseline always occurred in the morning on the day of surgery. As indicated in the timeline, the preoperative control and postoperative-3M blood samples were taken within a controlled timespan. B: baseline, preoperative; C: control, preoperative; E: effect, postoperative-3M, three months after surgery; Postoperative-6M: six months after surgical tumor resection, respectively.

All study participants were diagnosed with resectable stage I-IIIA NSCLC and underwent a lobectomy as the standard of care treatment. Demographics, clinical and pathological characteristics of the 74 study participants are summarized in Table 1. At the time of analysis, all patients were enrolled in the study during a follow-up period of at least six months after surgical resection of the tumor (postoperative-6M). It was clinically confirmed that none of the patients showed disease recurrence at the postoperative-6M time point. To investigate the effect of tumor removal on the metabolic response, all 74 study participants were asked to donate two preoperative and one postoperative fasted blood sample (Figure 2). The first blood sampling occurred after the diagnosis (median/range: 6/1–51 days) (control, C), and the second blood sample was taken on the morning of the day of surgery (baseline, B). The postoperative blood sampling occurred three months (median/range: 12/11–21 weeks) after surgical removal of the lung tumor (effect, E). All pre- and postoperative plasma samples were prepared and analyzed using a fixed protocol and quantitative ^1^H-NMR spectroscopy as described before [29]. Finally, a plasma metabolite profile was obtained at three specific time points for each patient. Accordingly, the metabolite profiles are defined by 228 well-defined integration regions in the ^1^H-NMR plasma spectrum and their integration values represent the concentration of 62 metabolites and are the variables for the multivariate statistics [29].

### 3.2. Baseline (B) versus Effect (E): Tumor Resection Causes a Shift in The Plasma Metabolite Profile of NSCLC Patients

**Figure 3:**
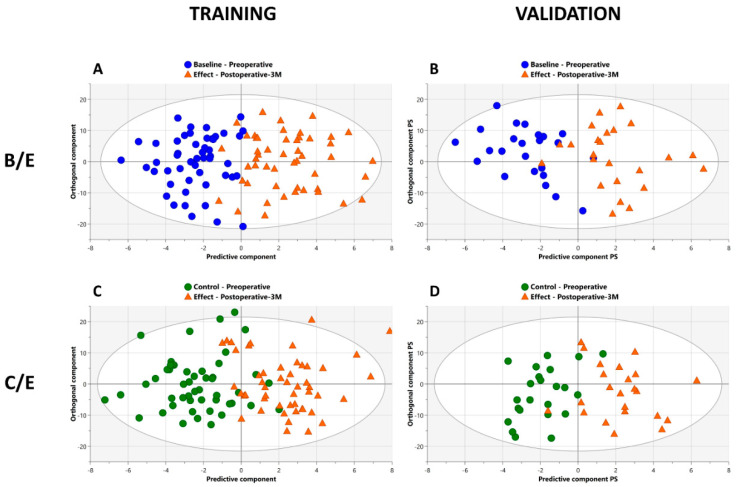
Detection of the metabolic shift between baseline/effect (**A**,**B**) and control/effect (**C**,**D**): the OPLS-DA classification models demonstrate the excellent separation between the pre- and postoperative plasma metabolite fingerprints of NSCLC patients. Each metabolite profile is represented by a specific labeled position in the OPLS-DA score plots. The clear separation on the predictive *x*-axis, representing the variation between the two groups, illustrates the metabolic shift between the pre- and postoperative metabolite profiles. (**A**) B/E training classification model (*s* = 100) using baseline preoperative and postoperative-3M plasma metabolic datasets of 50 NSCLC patients without early disease progression within six months after surgical tumor removal. The trained classifier allows excellent differentiation between the pre- and postoperative metabolite profile with 94% accuracy and shows great predictive accuracy, as demonstrated by the high Q2 value of 0.42. (**B**) B/E validation of classification (*s* = 48) based on the trained model using independent baseline preoperative and postoperative-3M plasma metabolic datasets of the remaining 24 patients. The validation confirms the observed metabolic shift between the pre- and postoperative metabolite profiles by discriminating with 90% accuracy between the two groups. (**C**) C/E training classification model (*s* = 100) using control preoperative and postoperative-3M plasma metabolic datasets of 50 NSCLC patients. The trained classifier confirms the clear differentiation between the pre- and postoperative metabolite profile with 89% accuracy, as shown in the B/E classifier. (**D**) C/E validation classification (*s* = 46) based on the trained model using independent control preoperative and postoperative-3M plasma metabolic datasets of the remaining 23 patients. Validation again confirms the observed metabolic shift between the pre- and postoperative metabolite profiles by showing a differentiation with 93% accuracy between the two groups. B: baseline, preoperative; C: control, preoperative; E: effect, postoperative-3M, three months after surgery; PS: predictive scores.

To evaluate the difference between the pre- and postoperative NSCLC metabolite profile, a supervised OPLS-DA model was first trained using the 228 variables from the baseline (B) and effect (E) datasets from 50 randomly selected patients (Figure 3A). This trained model allows excellent discrimination between the pre- and postoperative metabolite profiles, as demonstrated by a specificity of 96% (95% CI [86–100]) and a sensitivity of 92% (95% CI [81–98]). Furthermore, strong model parameters with an R2X and R2Y value of 0.55 and 0.67, combined with a high Q2 value of 0.42, confirm this model’s high predictive accuracy. In addition, a permutation test (500 permutations) resulted in a negative Q2 value of −0.43 in combination with an R2 value of 0.34, with a *p*-value < 0.001 (Figure S2). Such permutation test values combined with the absence of data overlap on the OPLS-DA predictive axis (x-axis) strongly support the strength of the trained model. Based on this trained classifier, an independent dataset of the remaining 24 patients was used for independent validation (Figure 3B), resulting in an excellent classification with 92% (95% CI [73–99]) specificity and 88% (95% CI [68–97]) sensitivity.

**Figure 4:**
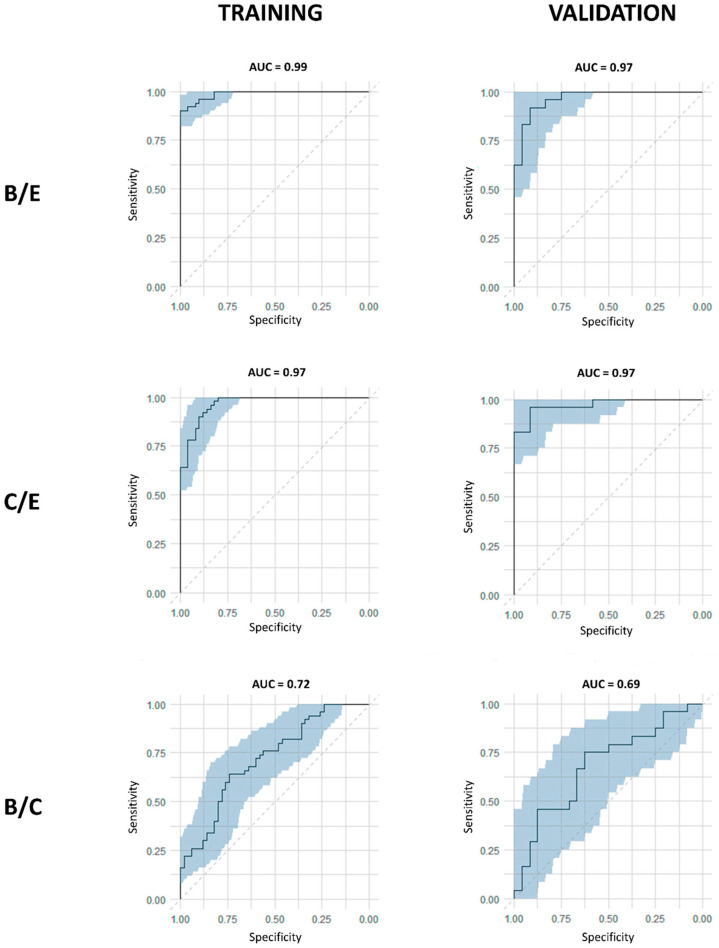
Receiving operating curves (ROC) demonstrate the excellent area under the curve (AUC) values for the differentiation between the pre- and postoperative B/E and C/E models. Cumulative ROC curves for the B/E and C/E OPLS-DA training and validation classification models show excellent AUC values in contrast to the ROC curves of the preoperative B/C OPLS-DA models. The gray zone represents the 95% confidence interval of the AUC values obtained by internal validation via bootstrapping resampling. AUC: area under the curve; B: baseline, preoperative; C: control, preoperative; E: effect, postoperative-3M, three months after surgery.

A more novel approach to analyze paired samples in a multivariate manner is OPLS-effect projections (OPLS-EP) [32]. By considering the dependency of the sample pairs (in this case: serial plasma samples of the same NSCLC patient) during the randomization process, improved model interpretations and the analysis of individual effects are obtained. Here, an OPLS-EP model for paired samples was constructed using the 228 variables. This model shows less complexity (less orthogonal axes) and an improved predictive ability (higher Q2 value) than the more traditional OPLS-DA analysis. The resulting R2X and R2Y values for this model were 0.59 and 0.89, respectively. Notably, this paired analysis’ excellent Q2 value of 0.76 strongly supports the difference between the pre- and postoperative metabolite profiles. In addition, as illustrated in Figure 4, the cumulative ROC curves demonstrate excellent AUC values of 0.99 (95% CI [0.98–1]) and 0.97 (95% CI [0.93–1]) for the trained and validation B/E model, respectively. Model parameters of all presented classifiers and ROC curves are summarized in Table 2.

### 3.3. Control (C) versus Effect (E): The Preoperative Metabolite Profile Can Be Determined at the Moment of NSCLC Diagnosis

Next, a second OPLS-DA model was trained to strengthen these findings by replacing the baseline dataset with the control (C) dataset. This trained C/E model also clearly separates the pre- and postoperative metabolite profiles with excellent model parameters for both OPLS-DA and OPLS-EP as summarized in Table 2 and demonstrated in Figure 3C. Furthermore, validating this trained model with an independent cohort of 23 patients (the same cohort of 24 patients as for the validation of the B/E model; one outlier excluded) results in a strong classification with a specificity of 91% (95% CI [72–99]) and a sensitivity of 96% (95% CI [78–100]) as demonstrated in Figure 3D.

### 3.4. Baseline (B) versus Control (C): The NSCLC Metabolite Profile Is Patient-Specific before Surgery

Extra confirmation of the impact of cancer on the metabolite profile can be found in modeling the baseline versus the control preoperative datasets from the same 50 patients. The model parameters of this B/C model are not only showing a substantially lower sensitivity and specificity but also a poor goodness of fit within (R2X) and between the groups (R2Y), as summarized in Table 2. The absence of the predictive ability of this model is clearly demonstrated by the very poor Q2 value of only 0.08. Furthermore, the absence of discriminating ability in the trained model is confirmed by the poor validation in the independent 23-patient cohort. The metabolite profiles at the two preoperative time points thus have to be quite similar and patient-specific.

### 3.5. Lactate, Cysteine, Asparagine and Acetate Are Identified as Key Contributors to the Metabolic Shift after NSCLC Surgery

**Figure 5:**
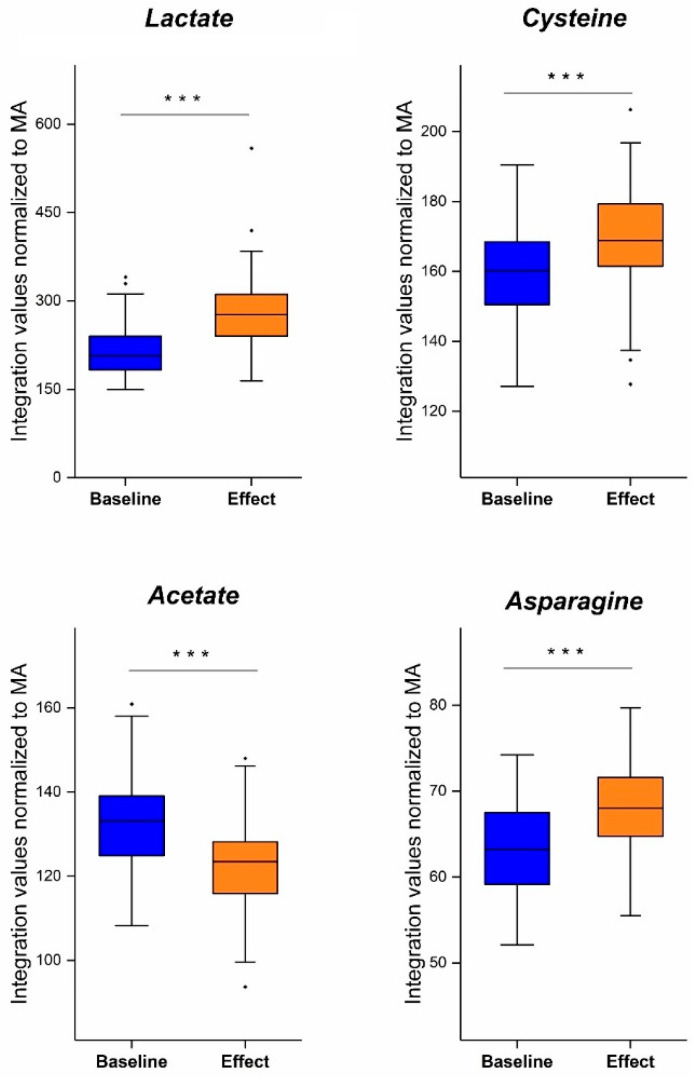
Boxplots showing the significant difference between the pre- and postoperative plasma concentration of lactate, cysteine, asparagine and acetate. Postoperative levels of lactate, cysteine and asparagine are elevated, while the postoperative acetate concentration is decreased. (***: significant results with *p*-value < 0.001, ·:datapoint outside the 1.5 IQR range).

The trained OPLS-DA (Table S1) and OPLS-EP (Table S2) B/E models were evaluated using VIP analysis to identify the variables that are the main contributors to the discrimination between the pre- and postoperative metabolite profile. S-plots were used to identify whether the variables show elevated or decreased concentrations The resulting tables showing the 30 most important variables are very comparable, meaning that both VIP analyses reveal the same variables as significant contributors in both the unpaired and paired classification model. Four metabolites corresponding to the proton signals of these 30 integration regions (i.e., the 30 integration regions corresponding to the 30 variables) were assigned based on metabolite spiking experiments (see also Section 2.5 and Table S4). Of these 30 variables, eleven variables correspond to proton signals that are highly selective for a single metabolite (proton signals that show no significant overlap with other signals): VAR 039, 040, 041 (lactate, quadruplet signal of ^α^CH); VAR 139, 140, 142, 143 (cysteine, double doublet signal of ^β^CH_2_); VAR 148, 150, 151 (asparagine, double doublet signal of ^β^CH_2_); VAR 199 (acetate, singlet signal of CH_3_) (Table S3). A normal distribution for each individual variable is indicated by the high *p*-values (>0.05) obtained from a Kolmogorov–Smirnov test (Table S3). Paired t-tests were used to compare the pre- and postoperative integration values of these proton signals. For lactate, cysteine and asparagine, the sum of the integration values of the selected metabolite-specific proton signals was used. The outcome reveals a postoperative increased concentration of lactate, cysteine and asparagine, together with a decreased level of acetate. Thus, both the unpaired and paired classification model uncover the importance of the same four metabolites during early NSCLC development. As shown in Figure 5, the pre- and postoperative levels of these four plasma metabolites are significantly different (*p*-value < 0.001).

## 4. Discussion

This prospective longitudinal trial demonstrates a significant shift in the metabolite plasma profile before and after the surgical resection of locally and early-advanced NSCLC. The excellent predictive accuracy of this metabolic shift is based on classifiers trained using two types of supervised multivariate analyses, i.e., OPLS-DA (R2X 0.55, R2Y 0.67, Q2 0.42) and OPLS-EP (R2X 0.59, R2Y 0.89, Q2 0.76) and validated with a sensitivity of 88% (95% CI [68–97]) and specificity of 92% (95% CI [73–99]) in an independent patient cohort. In addition, metabolite analysis reveals that the metabolic shift after NSCLC removal is characterized by a significant increase in the plasma levels of lactate, cysteine and asparagine together with a decrease in the plasma acetate concentration. Furthermore, the metabolic shift between the pre- and postoperative metabolite profiles indicates that the NSCLC metabolite profile is tumor-determined rather than donor-specific, as further confirmed by an additional OPLS-DA model with poor model parameters (R2X 0.31, R2Y 0.15, Q2 0.08) between the two preoperative time points. In conclusion, these results reveal that the metabolic difference caused by a tumor’s presence or absence is much larger than the individual’s daily metabolic variance.

One of the strengths of this study is the longitudinal design of plasma sampling at different time points. The large number of included patients with only locally and early-advanced resectable NSCLC allows randomly dividing the data into a training and independent validation set and results in reliable multivariate statistical discriminant analyses. However, longitudinal study designs have a high financial cost, are time-consuming and require intensive data management and multidisciplinary collaboration. For this reason, many reported studies are rather limited in the number of patients included. In this explorative study, the discriminative potential of pre- and postoperative plasma metabolite profiles is examined using 222 complete metabolic datasets obtained at three different time points from 74 NSCLC patients. Splitting the data into training (*s* = 100 for all trained models) and validation sets (*s* = 48 for B/E and *s* = 46 for C/E and B/C models) leaves both groups with a sufficient sample size for discriminative statistical analysis with adequate statistical power. However, no external validation using the data collected at different collaborating centers occurred yet in this sub-study.

A second important strength of this study is the use of a novel NMR-based methodology that provides substantial advantages for NMR metabolomics data analysis. NMR-metabolomics studies in human plasma are generally very complex and challenged by various complications such as binding of metabolites and internal standards to human serum albumin (HSA), patient-to-patient and sample-to-sample variation in HSA concentration, chemical shift fluctuations of metabolite signals, loss of statistical power due to peak fragmentation caused by shifting of the peaks beyond the limits of defined integration regions, and the absence of a reliable internal standard for normalization and quantification of signal intensities. The methodology as described by Derveaux et al. tackles these obstacles by the addition of (i) 4 mM TSP as a HSA-binding competitor and (ii) maleic acid (MA) as a reliable internal standard (i.e., in the presence of 4 mM TSP) [29]. The presence of 4 mM TSP eliminates the influence of sample-or patient-specific HSA concentration by saturating HSA with TSP, hereby preventing the binding of metabolites (and MA) to HSA, and resulting in the determination of accurate plasma metabolite concentrations. As another consequence, sample-to-sample differences in chemical shifts are eliminated (i.e., chemical shift dependency on the ratio HSA-bound/free metabolites is eliminated). Metabolite spiking experiments under the described experimental conditions provide a very accurate database consisting of 228 integration regions that, without chemical shift differences, provide a great statistical advantage for the univariate comparison of individual variables, as is performed in this study. Altogether, specific advantages of using this innovative methodology in combination with the large amount of plasma samples collected during the longitudinal study design (i.e., paired samples; three plasma samples from the same patient at specific timepoints), offer the possibility of using a specific workflow that solves important challenges in NMR-metabolomics and therefore also allows a specific statistical workflow for the analysis of the acquired data.

A limitation of the sub-study is the current lack of sufficient patient inclusions who show NSCLC recurrence after surgical resection. Of the 80 eligible patients, only 6 patients (=7.5%) were diagnosed with recurring NSCLC after six months, as shown in the study flow diagram in Figure 1. When more data from this patient group are available, the models presented in this work can directly be used for further discriminative analyses: adding the data of this patient group to the validation set might reveal a difference between the metabolite profiles of non-relapsing and relapsing patients. As a result, these analyses can strengthen the prospect of early-disease-recurrence detection via blood samples and might contribute to improving the stratification of patients during treatment follow-up. Introducing the concept of a responsive metabolic shift after NSCLC removal strongly supports the demand for further research. Further data collection is currently ongoing in the NCT03736993 trial. Next to increasing the current sample size at the three time points discussed in this work, other intermediate time points at four and six weeks after surgical resection will also be evaluated.

The use of fasted plasma samples is preferred over non-fasting samples as they were proven to have good reproducibility and minimize the impact of dietary habits [20,34]. Fasted blood sampling at two preoperative time points (baseline and control) provides important additional control and increases the reliability of the results. Furthermore, the fact that no predictive model can be constructed using data of the baseline and control time points of the same patients (Q2 value of only 0.08) demonstrates that the excellent discrimination between patients before and after surgery (Q2 value of 0.42 in OPLS-DA model and 0.76 in OPLS-EP model) is not caused by other effects than tumor removal and that the metabolite profiles are effectively cancer dependent.

Retrospective analysis of the clinical records of the three patients who were incorrectly classified in the B/E validation (Figure 3B) revealed that two of these patients had high C-reactive protein values at the time of postoperative blood sampling. An inflammatory profile might explain the wrong classification of these two postoperative metabolite profiles. Furthermore, a retrospective inspection of the initial diagnostic PET-CT images revealed a suspicious hypermetabolic colon lesion in the third patient. Based on the findings of this study, this patient has been referred for further diagnostic work up.

The data presented in this paper supports the findings of Ghini et al. They monitored 12 individuals over ten years and investigated the level of resilience, or metabolic flexibility, of the urine metabolite profile [18]. It was shown that, after a stress response, an individual’s metabolite profile returns to the profile inherently of that individual and remains unchanged for a long-term period. On the other hand, chronic stress responses, for example, caused by a tumor, seemed to induce an irreversible change during the timespan of the chronic condition. Our data strengthen the latter finding in a larger patient cohort by showing a changing metabolite profile after removing the chronic NSCLC stress condition.

Several long-term studies demonstrated that an individual’s metabolite fingerprint allows a correct subject identification based on a serial collection of samples during a long time interval in case of the absence of disease onset, indicating the donor-intrinsic character of the metabolic profile [11,18,19]. A study by Psychogios et al. revealed that the metabolite variability between individuals is larger than the longitudinal differences within a single individual [35]. Altogether, this means that if a metabolic shift is found, this shift overrules the variability observed between different individuals and can be considered a general driving force. When translated to this study that included only locally and early-advanced stage NSCLC patients, it appears that the presence or absence of a lung tumor (B/E and C/E) has a greater influence than patient-to-patient differences (B/C). Thus, it can be stated that the discriminating solid character of the B/E and C/E models is driven by the presence of cancer and not by patient-related factors. Or in short, the observed metabolic shift is caused by tumor removal. In conclusion, the ability to detect the metabolic difference before and after surgical tumor removal strongly supports the expectation that disease recurrence might also be detectable via blood samples. This ability indicates that the plasma metabolite profile shows excellent potential to serve as a minimally invasive real-time monitoring tool during therapy follow-up.

## 5. Conclusions

This study demonstrates that a responsive metabolic change occurs in locally and early-advanced NSCLC patients treated by surgical resection. Removing the tumor introduces a significant metabolic change in all 74 study participants who do not show cancer recurrence six months after surgery. This change is demonstrated by the successful differentiation between pre- and postoperative metabolite profiles with 92% specificity and 88% sensitivity. Furthermore, the metabolic shift is characterized by a significant increase in the levels of lactate, cysteine and asparagine, and decreased acetate in the postoperative plasma metabolite profile. The detection of this metabolic shift indicates the potential of the metabolite profile to detect early recurrence of NSCLC metabolism. Further patient inclusion and blood sample collection of NSCLC patients diagnosed with disease progression will provide more data to the constructed models and will further determine how the plasma metabolite profile can be used as a minimally invasive monitoring instrument during therapy follow-up of locally and early-advanced NSCLC patients.

## Figures and Tables

**Table 1:** Overview of the demographical, clinical and pathological characteristics of the 74 study participants diagnosed with resectable stage I-IIIA non-small-cell lung cancer. Pathological tumor staging was performed according to TNM classification, 8th edition. BMI: body mass index; COPD: chronic obstructive pulmonary disease; LVI: lymphovascular invasion; R0: negative resection margin; R1: positive resection margin with microscopic residual tumor; VPI: visceral pleural invasion.

		Training Cohort	Validation Cohort
Number of patients, *n*		50	24
Sex, *n* (%)	Male	27 (54)	15 (63)
	Female	23 (46)	9 (37)
Age, years (range)		68 ± 8 (45–83)	68 ± 9 (47–82)
BMI, kg/m^2^ (range)		26.9 ± 5.5 (16.6–50.4)	25.7 ± 3.8 (17.4–32.9)
Diabetes, *n* (%)		11 (22)	4 (17)
COPD, *n* (%)		11 (22)	5 (21)
Smoking status, *n* (%)	Active smoker	24 (48)	12 (50)
	Ex-smoker (stopped > 6 months)	25 (50)	10 (42)
	Non-smoker	1 (2)	2 (8)
Pathological tumor stage, *n* (%)	0	1 (2)	0 (0)
	IA1	3 (6)	1 (4)
	IA2	21 (42)	5 (21)
	IA3	7 (14)	5 (21)
	IB	5 (10)	7 (29)
	IIA	1 (2)	1 (4)
	IIB	6 (12)	0 (0)
	IIIA	6 (12)	5 (21)
Number of tumors, *n*		53	25
NSCLC tumor histology, *n* (%)	Adenocarcinoma	34 (64)	16 (64)
	Squamous carcinoma	13 (25)	8 (32)
	Adenosquamous carcinoma	1 (2)	0 (0)
	Neuroendocrine carcinoma	5 (9)	1 (4)
LVI, *n* (%)	Negative	44 (83)	17 (68)
	Positive	9 (17)	8 (32)
VPI, *n* (%)	Negative	45 (85)	18 (72)
	Positive	8 (15)	7 (28)
Resection margin, *n* (%)	R0	50 (94)	25 (100)
	R1	3 (6)	0 (0)

**Table 2:** Summary of the trained OPLS-DA and OPLS-EP model parameters with the resulting validation characteristics demonstrating a metabolic shift in NSCLC patients after surgical tumor resection. High Q2 values of the trained baseline/effect (B/E) and control/effect (C/E) classifiers indicate a good predictive accuracy, as confirmed by the validation with high specificity and sensitivity values, as well as an excellent differentiation between pre- and postoperative metabolite profiles of NSCLC patients. Modeling using the preoperative data (B/C) results as expected in very weak model parameters with a very poor predictive Q2 value and poor validation. These results are obtained from data of NSCLC patients that do not show disease recurrence within six months after surgery. OPLS-EP analysis takes the paired character of the samples into account. Using the same datasets reveals improved predictive accuracy of the trained classification models compared to OPLS-DA analysis. B: baseline, preoperative; C: control, preoperative; E: effect, postoperative-3M, three months after surgery; OPLS-DA: Orthogonal partial least squares discriminant analysis; OPLS-EP: OPLS-effect projections.

		B/E		C/E		B/C
		OPLS-DA	OPLS-EP	OPLS-DA	OPLS-EP	OPLS-DA
***TRAINING***						
	Number of patients, *n* (number of samples, *s*)	50 (100)	50 (100)	50 (100)	50 (100)	50 (100)
	R2X (cum)	0.55	0.59	0.53	0.57	0.31
	R2Y (cum)	0.67	0.89	0.61	0.83	0.15
	Q2 (cum)	0.42	0.76	0.36	0.60	0.08
	Specificity (%)	96 (86–100)	/	90 (78–97)	/	62 (47–75)
	Sensitivity (%)	92 (81–98)	/	88 (76–95)	/	74 (60–85)
	Accuracy (%)	94 (87–98)	86	89 (81–94)	84	68 (58–77)
	Fisher’s probability/ CV-ANOVA *p*-value	<0.001	<0.001	<0.001	<0.001	<0.001
	AUC	0.99 (0.98–1)	/	0.97 (0.94–1)	/	0.72 (0.62–0.82)
***VALIDATION***						
	Number of patients, *n* (number of samples, *s*)	24 (48)	24 (48)	23 (46)	23 (46)	23 (46)
	Specificity (%)	92 (73–99)	/	91 (72–99)	/	43 (23–66)
	Sensitivity (%)	88 (68–97)	/	96 (78–100)	/	74 (52–90)
	Accuracy (%)	90 (77–97)	92	93 (82–99)	87	59 (42–73)
	Fisher’s probability *p*-value	<0.001	/	<0.001	/	0.18
	AUC	0.97 (0.93–1)	/	0.97 (0.93–1)	/	0.69 (0.53–0.84)

## Data Availability

All clinical data were stored and monitored in Castor EDC, a GCP and GDPR-compliant database. In addition, an electronic case report form (eCRF) was created for each study participant. The first and corresponding author had full access to all the data. Supporting data for this study are available upon request.

## Supplementary Materials

The following supporting information can be downloaded at: https://www.mdpi.com/article/10.3390/cancers15072127/s1, Figure S1: One patient’s metabolite profile at the control time point is detected as a statistical outlier; Figure S2: Permutation test of the B/E trained OPLS-DA model using 500 permutations. Figure S3: PCA overview model coloured for tumor histology and overall pathological tumor staging. Table S1: Overview of the 30 variables with the highest VIP-values in the OPLS-DA model constructed with the 228 variables. Table S2: Overview of the 30 variables with the highest VIP-values in the OPLS-EP model constructed with the 228 variables. Table S3: Normality tests for the selected eleven variables. Table S4: Overview of the 228 variables (integration regions) and their corresponding metabolites.
