# Supplementary material for: NMR-Metabolomics Reveals a Metabolic Shift after Surgical Resection of Non-Small Cell Lung Cancer
Source: Cancers (Basel). 2023 Apr 3;15(7):2127. doi: 10.3390/cancers15072127 (PMC10093525; doi:10.3390/cancers15072127)
Supplement: Supplementary file 1 [file cancers-15-02127-s001.zip › cancers-2267306-supplementary.pdf]

SUPPLEMENTARY FIGURES

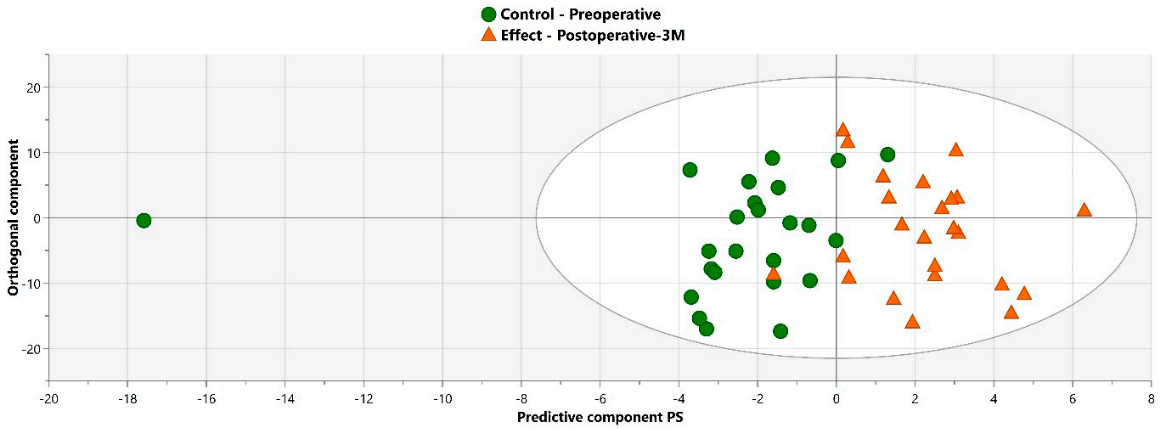

Figure S1: One patient's metabolite profile at the control time point is detected as a statistical outlier. By validating the B/E model using the validation cohort of 24 patients (*s* = 48), one patient's metabolite profile at the control time point is detected as a statistical outlier (green dot far outside of the white ellipse representing the 95% confidence interval of the validation model). For this reason, the atypical metabolite profile of the control time point and those of the matching effect and baseline time points were removed from the final analyses. Control, preoperative; E: Effect, postoperative-3M, three months after surgery; PS: predictive scores.

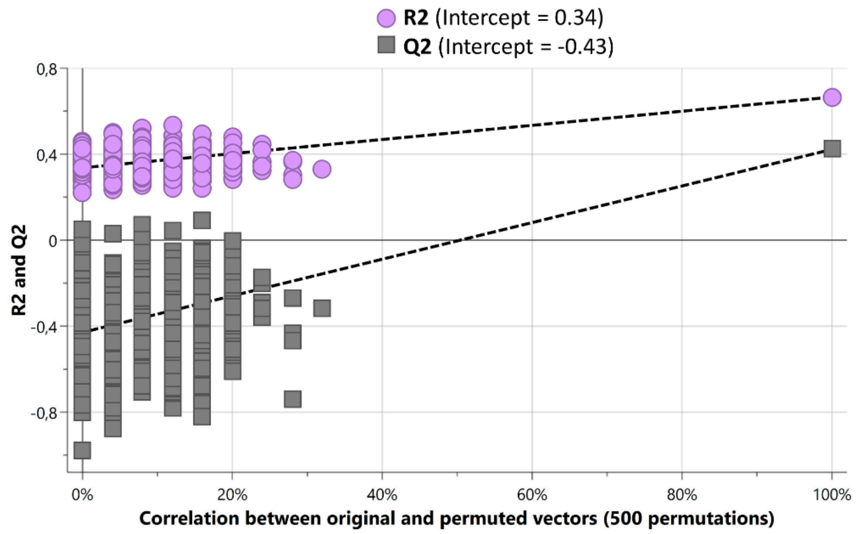

Figure S2: Permutation test of the B/E trained OPLS-DA model using 500 permutations. A negative Q2 value and R2X value > 0.20 of the permutation plot support the strong B/E classification model. The permutation test results in a p-value < 0.001.

A

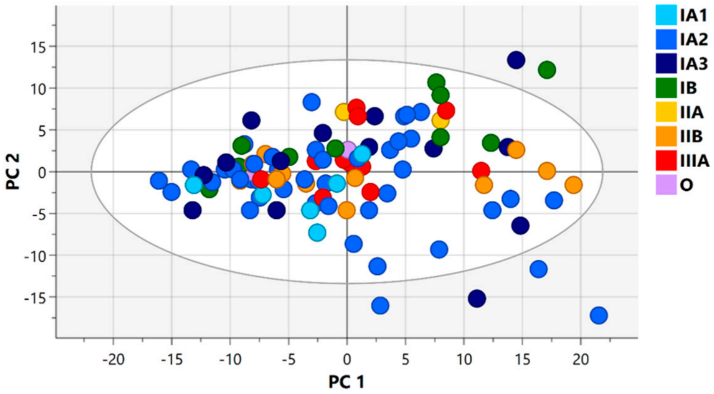

B

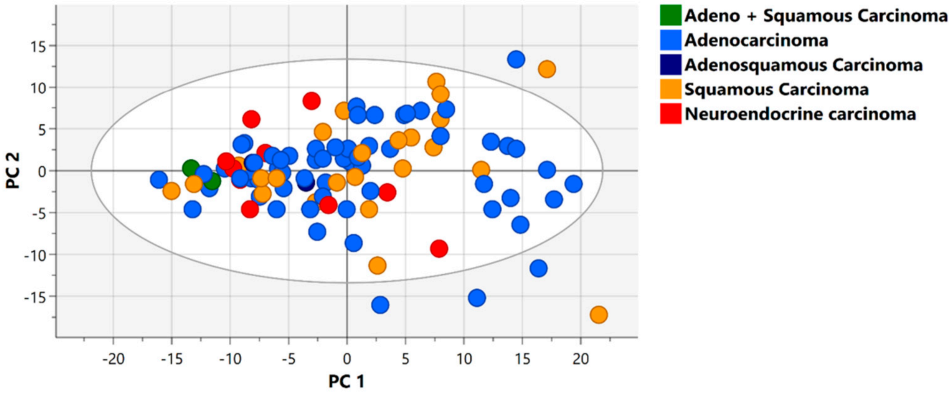

Figure S3: PCA model coloured for overall pathological tumor staging and tumor histology. The absence of clustering in the PCA plots indicates that neither pathological tumor stage (A) or tumor histology (B) are confounding variables associated with the observed separation between the pre-and postoperative metabolite profile. Pathological tumor staging was performed according to TNM classification, 8th edition.

SUPPLEMENTARY TABLES

Table S1: Overview of the 30 variables with the highest VIP-values in the OPLS-DA model constructed with the 228 variables representing the pre-and postoperative metabolite profile of 50 NSCLC patients: a high VIP-value indicates the importance of the variable, and so its representing metabolite, to the metabolic differentiation of the pre-and postoperative metabolite profiles. Green and red arrows indicate increased and decreased integration values in the postoperative compared to the preoperative metabolite profile. VAR: variable (integration value of the integration region as listed in table S4); VIP: variable importance for the projection.

Variable number	Total VIP-value	Variable number	Total VIP-value
**VAR 040**	2.77 ↑	**VAR 159**	1.50 ↑
**VAR 041**	2.57 ↑	**VAR 146**	1.48 ↑
**VAR 039**	2.54 ↑	**VAR 101**	1.48 ↓
**VAR 199**	1.96 ↓	**VAR 145**	1.46 ↑
**VAR 139**	1.86 ↑	**VAR 083**	1.46 ↑
**VAR 138**	1.82 ↑	**VAR 091**	1.46 ↑
**VAR 136**	1.78 ↑	**VAR 068**	1.46 ↑
**VAR 215**	1.78 ↑	**VAR 152**	1.40 ↑
**VAR 137**	1.75 ↑	**VAR 082**	1.36 ↑
**VAR 167**	1.74 ↑	**VAR 002**	1.35 ↑
**VAR 150**	1.65 ↑	**VAR 169**	1.34 ↑
**VAR 148**	1.62 ↑	**VAR 130**	1.32 ↑
**VAR 149**	1.56 ↑	**VAR 102**	1.29 ↓
**VAR 151**	1.54 ↑	**VAR 132**	1.28 ↑
**VAR 143**	1.51 ↑	**VAR 128**	1.27 ↑

Table S2: Overview of the 30 variables with the highest VIP-values in the OPLS-EP model constructed with the 228 variables representing the difference between the pre-and postoperative metabolite profile of 50 NSCLC patients: a high VIP-value indicates the importance of the variable, and so its representing metabolite, to the metabolic differentiation of the pre-and postoperative metabolite profiles. Green and red arrows indicate increased and decreased integration values in the postoperative compared to the preoperative metabolite profile. VAR: variable (integration value of the integration region as listed in table S4); VIP: variable importance for the projection.

Variable number	Total VIP-value	Variable number	Total VIP-value
**VAR 040**	2.11 ↑	**VAR 148**	1.45 ↑
**VAR 039**	2.05 ↑	**VAR 152**	1.45 ↑
**VAR 041**	2.04 ↑	**VAR 101**	1.45 ↓
**VAR 139**	2.02 ↑	**VAR 150**	1.44 ↑
**VAR 215**	1.83 ↑	**VAR 102**	1.42 ↓
**VAR 138**	1.75 ↑	**VAR 158**	1.37 ↑
**VAR 199**	1.75 ↓	**VAR 149**	1.37 ↑
**VAR 159**	1.74 ↑	**VAR 091**	1.37 ↑

**VAR 140**	1.70 ↑	**VAR 145**	1.36 ↑
**VAR 167**	1.64 ↑	**VAR 146**	1.36 ↑
**VAR 092**	1.57 ↑	**VAR 128**	1.34 ↑
**VAR 137**	1.57 ↑	**VAR 083**	1.32 ↑
**VAR 151**	1.52 ↑	**VAR 160**	1.32 ↑
**VAR 143**	1.47 ↑	**VAR 068**	1.32 ↑
**VAR 136**	1.46 ↑	**VAR 178**	1.28 ↑

Table S3: Normality test for the eleven selected variables. The Kolmogorov-Smirnov test is used for normality testing of each variable. P-values > 0.05 indicate that all eleven variables are normally distributed. dd: double doublet; s: singlet; q: quadruplet. VAR: variable (integration value of the integration region as listed in table S4).

**Selected variable**	**K-S Normality test**				**Assigned metabolite**	**Corresponding proton signal and multiplicity**
	**Baseline**		**Effect**			
	K-S test statistic	P-value	K-S test statistic	P-value		
**VAR 039**	0.092	0.541	0.073	0.862	Lactate	$^{\alpha}$CH (q)
**VAR 040**	0.094	0.513	0.093	0.517	Lactate	$^{\alpha}$CH (q)
**VAR 041**	0.116	0.251	0.726	0.726	Lactate	$^{\alpha}$CH (q)
**VAR 139**	0.096	0.486	0.119	0.225	Cysteine	$^{\beta}CH_2$ (dd)
**VAR 140**	0.072	0.894	0.133	0.131	Cysteine	$^{\beta}CH_2$ (dd)
**VAR 142**	0.093	0.520	0.060	1.000	Cysteine	$^{\beta}CH_2$ (dd)
**VAR 143**	0.089	0.589	0.095	0.490	Cysteine	$^{\beta}CH_2$ (dd)
**VAR 148**	0.083	0.686	0.050	1.000	Asparagine	$^{\beta}CH_2$ (dd)
**VAR 150**	0.052	1.000	0.076	0.803	Asparagine	$^{\beta}CH_2$ (dd)
**VAR 151**	0.085	0.648	0.063	1.000	Asparagine	$^{\beta}CH_2$ (dd)
**VAR 199**	0.060	1.000	0.066	0.996	Acetate	CH_3 (s)

Table S4, as published by Derveaux et al (2021). Overview of the used 228 integration regions (VAR-number) of which the 228 integration values = variables, are used in the statistical analyses and their corresponding metabolites. All integration regions and corresponding metabolites are assigned based on spiking experiments. Nine variables, indicated with *, of the initial list of 237 integration regions) are not used. FAC: fatty acid chain; NAG: N-acetylated glycoproteins; NI: non-identified; PC: phosphatidylcholine; PL: phospholipids; SM: sphingomyelin; TG: triglycerides;

	**Variable number**	**Integration region (ppm)**	**Corresponding metabolite(s)**
*	**VAR 001**	**8.460 – 8.484**	Formate
	**VAR 002**	**8.190 – 8.240**	Hypoxanthine
	**VAR 003**	**7.945 – 8.170**	NI 1
	**VAR 004**	**7.905 – 7.945**	1-methylhistidine
	**VAR 005**	**7.875 – 7.905**	Uridine
	**VAR 006**	**7.845 – 7.875**	Histidine
	**VAR 007**	**7.820 – 7.845**	NI 2

	**VAR 008**	**7.720 – 7.780**	Tryptophan<br>NI 3
*	**VAR 009**	**7.650 - 7.700**	3-methylhistidine
	**VAR 010**	**7.530 - 7.580**	Tryptophan
	**VAR 011**	**7.350 - 7.480**	Phenylalanine
	**VAR 012**	**7.330 - 7.350**	Phenylalanine<br>Tryptophan
	**VAR 013**	**7.260 - 7.320**	Tryptophan<br>NI 4
	**VAR 014**	**7.240 - 7.260**	NI 5
	**VAR 015**	**7.180 - 7.240**	Tryptophan<br>Tyrosine
	**VAR 016**	**7.080 - 7.140**	Histidine
	**VAR 017**	**6.998 - 7.071**	1-methylhistidine<br>3-methylhistidine<br>NI 6
	**VAR 018**	**6.954 - 6.986**	NI 7
	**VAR 019**	**6.890 - 6.950**	Tyrosine
	**VAR 020**	**6.730 - 6.775**	NI 8
*	**VAR 021**	**6.533 - 6.547**	Fumarate
*	**VAR 022**	**5.907 - 5.946**	Uridine
	**VAR 023**	**5.720 - 5.865**	NI 9
*	**VAR 024**	**5.405 - 5.415**	Allantoin
	**VAR 025**	**5.238 - 5.267**	Glucose
	**VAR 026**	**5.196 - 5.210**	Mannose
*	**VAR 027**	**4.919 - 4.928**	Mannose
	**VAR 028**	**4.633 - 4.703**	Glucose<br>Hydroxyproline<br>NI 10
*	**VAR 029**	**4.560 - 4.614**	Carnitine
*	**VAR 030**	**4.528 - 4.540**	NI 11
*	**VAR 031**	**4.521 - 4.527**	NI 12
	**VAR 032**	**4.450 - 4.490**	NI 13
	**VAR 033**	**4.347 - 4.430**	Hydroxyproline<br>N-acetylcysteine<br>Uridine<br>Lipids: C_1H and C_3H in glycerol backbone of PL and TG
	**VAR 034**	**4.299 - 4.347**	Lipids: (O-C**H_2**-CH_2-$N^+(CH_3)_3$ of PC and SM; C_1H and C_3H in glycerol backbone of PL and TG
	**VAR 035**	**4.269 - 4.299**	Threonine<br>Lipids: (O-C**H_2**-CH_2-$N^+(CH_3)_3$ of PC and SM
	**VAR 036**	**4.237 - 4.269**	Threonine<br>Uridine

**VAR 037**	**4.172 - 4.216**	Pyroglutamate<br>β-Hydroxybutyrate
**VAR 038**	**4.153 - 4.172**	Proline<br>Uridine<br>β-Hydroxybutyrate
**VAR 039**	**4.147 - 4.153**	Lactate<br>Proline<br>Uridine
**VAR 040**	**4.133 - 4.147**	Lactate<br>Proline<br>Uridine<br>β-Hydroxybutyrate<br>Cystine
**VAR 041**	**4.110 - 4.133**	Lactate<br>Cystine
**VAR 042**	**4.054 - 4.102**	Choline<br>Creatinine<br>Myoinositol<br>Tryptophan<br>Isopropanol<br>NI 14
**VAR 043**	**4.043 - 4.054**	Isopropanol<br>NI 14
**VAR 044**	**4.034 - 4.043**	Isopropanol
**VAR 045**	**4.021 - 4.034**	2-hydroxybutyrate<br>Asparagine<br>Phenylalanine<br>Serine<br>Isopropanol
**VAR 046**	**4.006 - 4.021**	2-hydroxybutyrate<br>Asparagine<br>Histidine<br>Phenylalanine<br>Serine<br>Isopropanol
**VAR 047**	**4.001 - 4.006**	Histidine<br>Phenylalanine<br>Serine
**VAR 048**	**3.993 - 4.001**	3-methylhistidine<br>Histidine<br>Serine
**VAR 049**	**3.985 - 3.992**	3-methylhistidine<br>Cysteine

**VAR 050**	**3.979 - 3.985**	3-methylhistidine<br>Cysteine<br>Serine
**VAR 051**	**3.968 - 3.979**	1-methylhistidine<br>3-methylhistidine<br>Cysteine<br>Mannose<br>Serine<br>Tyrosine
**VAR 052**	**3.960 - 3.968**	1-methylhistidine<br>Mannose<br>Serine<br>Tyrosine
**VAR 053**	**3.954 - 3.960**	1-methylhistidine<br>Mannose<br>Tyrosine
**VAR 054**	**3.946 - 3.954**	1-methylhistidine<br>Creatine<br>Mannose<br>Serine
**VAR 055**	**3.934 - 3.946**	Mannose<br>Uridine
**VAR 056**	**3.930 - 3.934**	Aspartate<br>Glucose
**VAR 057**	**3.914 - 3.930**	Aspartate<br>Betaine<br>Glucose<br>Mannose<br>Uridine
**VAR 058**	**3.909 - 3.914**	Aspartate<br>Glucose
**VAR 059**	**3.900 - 3.909**	Glucose<br>Mannose<br>NI 15
**VAR 060**	**3.893 - 3.900**	NI 15
**VAR 061**	**3.885 - 3.893**	Mannose<br>Methionine
**VAR 062**	**3.873 - 3.885**	Glucose<br>Mannose<br>Methionine<br>NI 15
**VAR 063**	**3.857 - 3.873**	2-hydroxy-3-methylbutyrate<br>Glucose

		Mannose<br>Methionine<br>Serine
**VAR 064**	**3.851 - 3.857**	Glucose<br>Mannose<br>Serine
**VAR 065**	**3.846 - 3.851**	Glucose<br>Mannose
**VAR 066**	**3.829 - 3.844**	Glucose<br>Mannose<br>Uridine
**VAR 067**	**3.818 - 3.829**	Alanine<br>Mannose<br>Uridine<br>Glycerol
**VAR 068**	**3.808 - 3.818**	Alanine<br>Ornithine<br>Uridine<br>Glycerol
**VAR 069**	**3.796 - 3.808**	Alanine<br>Arginine<br>Glucose<br>Glutamine<br>Mannose<br>Ornithine<br>Glycerol
**VAR 070**	**3.785 - 3.796**	Alanine<br>Arginine<br>Glucose<br>Glutamate<br>Glutamine<br>Lysine<br>Mannose<br>Ornithine<br>Glycerol
**VAR 071**	**3.770 - 3.785**	Arginine<br>Glucose<br>Glutamate<br>Glutamine<br>Lysine<br>Mannose
**VAR 072**	**3.765 - 3.770**	Glutamate<br>Leucine<br>Lysine

**VAR 073**	**3.748 - 3.765**	Glucose<br>Leucine<br>Mannose
**VAR 074**	**3.739 - 3.748**	Glucose<br>Leucine<br>Mannose<br>2-aminobutyrate
**VAR 075**	**3.728 - 3.739**	Glucose<br>2-aminobutyrate
**VAR 076**	**3.715 - 3.728**	1-methylhistidine<br>Glucose<br>2-aminobutyrate<br>NI 16
**VAR 077**	**3.709 - 3.713**	3-methylhistidine
**VAR 078**	**3.704 - 3.709**	NI 16
**VAR 079**	**3.685 - 3.704**	Isoleucine<br>Mannose<br>Glycerol<br>NI 16<br>NI 17<br>Lipids: (O-CH_2-C**H_2**-$N^+(CH_3)_3$ of PC and SM
**VAR 080**	**3.678 - 3.685**	Mannose<br>Glycerol<br>NI 17<br>Lipids: (O-CH_2-C**H_2**-$N^+(CH_3)_3$ of PC and SM
**VAR 081**	**3.673 - 3.678**	Mannose<br>Lipids: (O-CH_2-C**H_2**-$N^+(CH_3)_3$ of PC and SM
**VAR 082**	**3.664 - 3.673**	Mannose<br>Glycerol<br>NI 17<br>Lipids: (O-CH_2-C**H_2**-$N^+(CH_3)_3$ of PC and SM
**VAR 083**	**3.654 - 3.664**	Myoinositol<br>Glycerol<br>NI 17<br>Lipids: (O-CH_2-C**H_2**-$N^+(CH_3)_3$ of PC and SM
**VAR 084**	**3.649 - 3.654**	Lipids: (O-CH_2-C**H_2**-$N^+(CH_3)_3$ of PC and SM
**VAR 085**	**3.642 - 3.647**	Myoinositol
**VAR 086**	**3.633 - 3.641**	Valine
**VAR 087**	**3.623 - 3.633**	Myoinositol<br>Sarcosine<br>Valine
**VAR 088**	**3.608 - 3.616**	Mannose<br>Threonine

**VAR 089**	**3.600 - 3.606**	Threonine
**VAR 090**	**3.594 - 3.600**	Mannose<br>Glycerol
**VAR 091**	**3.584 - 3.589**	Glycerol
**VAR 092**	**3.576 - 3.584**	Glycine<br>Mannose<br>Glycerol
**VAR 093**	**3.559 - 3.572**	Glucose<br>Myoinositol<br>Glycerol
**VAR 094**	**3.541 - 3.555**	Choline<br>Glucose<br>Myoinositol
**VAR 095**	**3.534 - 3.541**	Choline
**VAR 096**	**3.524 - 3.534**	Choline<br>Glucose
**VAR 097**	**3.507 - 3.522**	Glucose<br>Hydroxyproline<br>Tryptophan
**VAR 098**	**3.500 - 3.504**	Glucose<br>Tryptophan
**VAR 099**	**3.490 - 3.500**	Glucose<br>Hydroxyproline<br>Tryptophan
**VAR 100**	**3.467 - 3.490**	Glucose<br>Tryptophan
**VAR 101**	**3.460 - 3.467**	Proline<br>Acetoacetate
**VAR 102**	**3.455 - 3.460**	Carnitine
**VAR 103**	**3.437 - 3.455**	Carnitine<br>Glucose<br>Proline<br>Taurine
**VAR 104**	**3.433 - 3.437**	Glucose
**VAR 105**	**3.429 - 3.433**	Proline<br>Taurine
**VAR 106**	**3.413 - 3.429**	Mannose<br>Glucose<br>Proline<br>Cystine
**VAR 107**	**3.401 - 3.413**	Glucose<br>Mannose
**VAR 108**	**3.387 - 3.401**	Hydroxyproline

		Mannose<br>Cystine
**VAR 109**	**3.383 - 3.387**	Hydroxyproline
**VAR 110**	**3.375 - 3.383**	Hydroxyproline<br>Proline
**VAR 111**	**3.354 - 3.373**	Proline
**VAR 112**	**3.346 - 3.352**	Proline<br>Tryptophan
**VAR 113**	**3.332 - 3.340**	1-methylhistidine<br>Proline<br>Tryptophan
**VAR 114**	**3.313 - 3.325**	1-methylhistidine<br>Myoinositol<br>Phenylalanine<br>Tryptophan
**VAR 115**	**3.304 - 3.313**	1-methylhistidine<br>Phenylalanine<br>Tryptophan
**VAR 116**	**3.300 - 3.304**	Myoinositol
**VAR 117**	**3.291 - 3.300**	1-methylhistidine<br>Phenylalanine<br>Taurine<br>Lipids: (O-CH_2-CH_2-N^+(C$\mathbf{H_3}$)$_3$ of PC and SM
**VAR 118**	**3.284 - 3.289**	Betaine<br>Myoinositol<br>Phenylalanine<br>Lipids: (O-CH_2-CH_2-N^+(C$\mathbf{H_3}$)$_3$ of PC and SM
**VAR 119**	**3.278 - 3.284**	Glucose<br>Histidine<br>Taurine<br>Lipids: (O-CH_2-CH_2-N^+(C$\mathbf{H_3}$)$_3$ of PC and SM
**VAR 120**	**3.270 - 3.278**	Arginine<br>Histidine<br>Taurine<br>Lipids: (O-CH_2-CH_2-N^+(C$\mathbf{H_3}$)$_3$ of PC and SM
**VAR 121**	**3.263 - 3.270**	Arginine<br>Glucose<br>Lipids: (O-CH_2-CH_2-N^+(C$\mathbf{H_3}$)$_3$ of PC and SM
**VAR 122**	**3.250 - 3.263**	Arginine<br>Glucose<br>Histidine<br>Lipids: (O-CH_2-CH_2-N^+(C$\mathbf{H_3}$)$_3$ of PC and SM
**VAR 123**	**3.244 - 3.250**	1-methylhistidine

		Carnitine<br>Histidine<br>Lipids: (O-CH_2-CH_2-N^+(C**H₃**)$_3$ of PC and SM
**VAR 124**	**3.236 - 3.244**	Lipids: (O-CH_2-CH_2-N^+(CH_3)$_3$ of PC and SM
**VAR 125**	**3.231 - 3.236**	1-methylhistidine<br>Tyrosine<br>Lipids: (O-CH_2-CH_2-N^+(CH_3)$_3$ of PC and SM
**VAR 126**	**3.227 - 3.231**	Cystine<br>Lipids: (O-CH_2-CH_2-N^+(CH_3)$_3$ of PC and SM
**VAR 127**	**3.224 - 3.227**	Tyrosine<br>Lipids: (O-CH_2-CH_2-N^+(CH_3)$_3$ of PC and SM
**VAR 128**	**3.217 - 3.222**	1-methylhistidine<br>Choline<br>Tyrosine<br>Lipids: (O-CH_2-CH_2-N^+(CH_3)$_3$ of PC and SM
**VAR 129**	**3.211 - 3.217**	β-alanine<br>Tyrosine<br>Cystine<br>Lipids: (O-CH_2-CH_2-N^+(CH_3)$_3$ of PC and SM
**VAR 130**	**3.195 - 3.211**	1-methylhistidine<br>3-methylhistidine<br>β-alanine<br>Tyrosine<br>Cystine<br>Lipids: (O-CH_2-CH_2-N^+(CH_3)$_3$ of PC and SM
**VAR 131**	**3.187 - 3.193**	β-alanine<br>Cystine
**VAR 132**	**3.177 - 3.183**	3-methylhistidine<br>Histidine
**VAR 133**	**3.169 - 3.175**	3-methylhistidine
**VAR 134**	**3.135 - 3.169**	Histidine<br>Phenylalanine
**VAR 135**	**3.122 - 3.134**	Cysteine<br>Phenylalanine
**VAR 136**	**3.100 - 3.122**	3-methylhistidine<br>Cysteine
**VAR 137**	**3.090 - 3.100**	3-methylhistidine<br>Cysteine<br>Tyrosine
**VAR 138**	**3.077 - 3.090**	3-methyl-2-oxobutyrate<br>3-methylhistidine<br>Ornithine<br>Tyrosine

**VAR 139**	**3.066 - 3.077**	3-methyl-2-oxobutyrate<br>3-methylhistidine<br>Cysteine<br>Ornithine<br>Tyrosine
**VAR 140**	**3.060 - 3.066**	3-methyl-2-oxobutyrate<br>Creatinine<br>Cysteine<br>Ornithine
**VAR 141**	**3.053 - 3.060**	3-methyl-2-oxobutyrate<br>Creatine<br>Lysine<br>Tyrosine
**VAR 142**	**3.040 - 3.053**	3-methyl-2-oxobutyrate<br>4-aminobutyrate<br>Cysteine<br>Lysine
**VAR 143**	**3.035 - 3.040**	3-methyl-2-oxobutyrate<br>Cysteine<br>α-ketoglutarate
**VAR 144**	**3.029 - 3.035**	4-aminobutyrate<br>Lysine
**VAR 145**	**3.023 - 3.029**	3-methyl-2-oxobutyrate<br>α-ketoglutarate
**VAR 146**	**3.017 - 3.023**	4-aminobutyrate
**VAR 147**	**3.010 - 3.017**	α-ketoglutarate
**VAR 148**	**2.979 - 2.991**	Asparagine
**VAR 149**	**2.966 - 2.979**	3-methyl-2-oxopentanoate<br>N-acetylcysteine
**VAR 150**	**2.958 - 2.966**	3-methyl-2-oxopentanoate<br>Asparagine
**VAR 151**	**2.950 - 2.958**	Asparagine<br>N-acetylcysteine
**VAR 152**	**2.911 - 2.950**	3-methyl-2-oxopentanoate<br>N-acetylcysteine
**VAR 153**	**2.875 - 2.904**	Asparagine
**VAR 154**	**2.854 - 2.875**	Asparagine<br>Lipids: =CH-C$\mathbf{H}_2$-CH= in FAC
**VAR 155**	**2.812 - 2.854**	Aspartate<br>Lipids: =CH-C$\mathbf{H}_2$-CH= in FAC
**VAR 156**	**2.762 - 2.812**	Lipids: =CH-C$\mathbf{H}_2$-CH= in FAC
**VAR 157**	**2.757 - 2.762**	Sarcosine<br>Lipids: =CH-C$\mathbf{H}_2$-CH= in FAC

**VAR 158**	**2.741 - 2.757**	Lipids: =CH-C**H_2**-CH= in FAC
**VAR 159**	**2.724 - 2.741**	NI 18<br>Lipids: =CH-C**H_2**-CH= in FAC
**VAR 160**	**2.718 - 2.724**	Aspartate<br>Lipids: =CH-C**H_2**-CH= in FAC
**VAR 161**	**2.712 - 2.718**	Lipids: =CH-C**H_2**-CH= in FAC
**VAR 162**	**2.702 - 2.712**	Aspartate<br>Citrate
**VAR 163**	**2.690 - 2.694**	Aspartate
**VAR 164**	**2.673 - 2.685**	Aspartate<br>Citrate<br>Methionine
**VAR 165**	**2.647 - 2.668**	Methionine
**VAR 166**	**2.615 - 2.634**	4-methyl-2-oxovalerate
**VAR 167**	**2.571 - 2.592**	β-alanine
**VAR 168**	**2.533 - 2.571**	β-alanine<br>Citrate<br>Pyroglutamate
**VAR 169**	**2.501 - 2.533**	Glutamine<br>Pyroglutamate
**VAR 170**	**2.484 - 2.501**	Carnitine<br>Glutamine<br>Pyroglutamate
**VAR 171**	**2.476 - 2.484**	Glutamine
**VAR 172**	**2.450 - 2.476**	Carnitine<br>Glutamine<br>Hydroxyproline<br>α-ketoglutarate
**VAR 173**	**2.439 - 2.450**	Carnitine<br>Glutamine<br>Hydroxyproline<br>α-ketoglutarate<br>β-Hydroxybutyrate
**VAR 174**	**2.427 - 2.439**	Carnitine<br>Glutamine<br>Hydroxyproline<br>Pyroglutamate<br>β-Hydroxybutyrate
**VAR 175**	**2.418 - 2.427**	Carnitine<br>Pyroglutamate<br>Succinate<br>β-Hydroxybutyrate
**VAR 176**	**2.405 - 2.418**	Glutamate

		Pyroglutamate<br>β-Hydroxybutyrate
**VAR 177**	**2.380 - 2.405**	3-hydroxy-3-methylbutyrate<br>Glutamate<br>Oxaloacetate<br>Proline<br>Pyruvate
**VAR 178**	**2.349 - 2.380**	Glutamate<br>Proline
**VAR 179**	**2.337 - 2.349**	Glutamate<br>Proline<br>β-Hydroxybutyrate
**VAR 180**	**2.324 - 2.337**	4-aminobutyrate<br>Glutamate<br>Valine<br>β-Hydroxybutyrate
**VAR 181**	**2.312 - 2.324**	4-aminobutyrate<br>Valine<br>β-Hydroxybutyrate
**VAR 182**	**2.305 - 2.312**	Valine<br>β-Hydroxybutyrate
**VAR 183**	**2.294 - 2.305**	4-aminobutyrate<br>Valine<br>Acetoacetate
**VAR 184**	**2.282 - 2.294**	Valine
**VAR 185**	**2.264 - 2.282**	Valine<br>Lipids: -C**H_2**-C=O or -C**H_2**-CH=CH- in FAC
**VAR 186**	**2.254 - 2.264**	Lipids: -C**H_2**-C=O or -C**H_2**-CH=CH- in FAC
**VAR 187**	**2.221 - 2.254**	Methionine<br>Lipids: -C**H_2**-C=O or -C**H_2**-CH=CH- in FAC
**VAR 188**	**2.208 - 2.221**	Methionine
**VAR 189**	**2.151 - 2.208**	Glutamate<br>Glutamine<br>Hydroxyproline<br>Methionine
**VAR 190**	**2.128 - 2.151**	4-methyl-2-oxovalerate<br>Glutamate<br>Glutamine<br>Methionine
**VAR 191**	**2.114 - 2.128**	4-methyl-2-oxovalerate<br>Glutamate<br>Glutamine<br>Methionine

		Proline
**VAR 192**	**2.102 - 2.114**	4-methyl-2-oxovalerate<br>Glutamate<br>Glutamine<br>Methionine<br>Proline<br>Lipids: -C**H_2**-CH=CH- in FAC; C**H_3** of NAG
**VAR 193**	**2.078 - 2.102**	Glutamate<br>N-acetylcysteine<br>Proline<br>Pyroglutamate<br>Lipids: -C**H_2**-CH=CH- in FAC; C**H_3** of NAG
**VAR 194**	**2.042 - 2.078**	2-hydroxy-3-methylbutyrate<br>Glutamate<br>Proline<br>Pyroglutamate<br>Lipids: -C**H_2**-CH=CH- in FAC; C**H_3** of NAG
**VAR 195**	**2.013 - 2.042**	2-hydroxy-3-methylbutyrate<br>Isoleucine<br>Proline<br>Pyroglutamate<br>Lipids: -C**H_2**-CH=CH- in FAC; C**H_3** of NAG
**VAR 196**	**2.001 - 2.013**	Isoleucine<br>Ornithine<br>Proline<br>Lipids: -C**H_2**-CH=CH- in FAC; C**H_3** of NAG
**VAR 197**	**1.967 - 2.001**	Arginine<br>Isoleucine<br>Ornithine<br>Proline<br>2-aminobutyrate<br>Lipids: -C**H_2**-CH=CH- in FAC; C**H_3** of NAG
**VAR 198**	**1.949 - 1.967**	Arginine<br>Lysine<br>Ornithine<br>2-aminobutyrate
**VAR 199**	**1.906 - 1.949**	4-aminobutyrate<br>Acetate<br>Arginine<br>Lysine<br>Ornithine<br>2-aminobutyrate

**VAR 200**	**1.823 - 1.906**	4-aminobutyrate<br>Arginine<br>Lysine<br>Ornithine<br>2-aminobutyrate
**VAR 201**	**1.800 - 1.823**	Ornithine
**VAR 202**	**1.729 - 1.800**	2-hydroxybutyrate<br>3-methyl-2-oxopentanoate<br>Arginine<br>Leucine<br>Lysine<br>Ornithine
**VAR 203**	**1.717 - 1.729**	2-hydroxybutyrate<br>3-methyl-2-oxopentanoate<br>Arginine<br>Leucine<br>Lysine
**VAR 204**	**1.678 - 1.717**	2-hydroxybutyrate<br>3-methyl-2-oxopentanoate<br>Arginine<br>Leucine
**VAR 205**	**1.655 - 1.678**	2-hydroxybutyrate<br>Arginine
**VAR 206**	**1.650 - 1.655**	Arginine
**VAR 207**	**1.630 - 1.650**	2-hydroxybutyrate<br>Arginine<br>Lipids: -C**H$_2$**-CH$_2$-C=O or -C**H$_2$**-CH$_2$-CH=CH- in FAC
**VAR 208**	**1.572 - 1.630**	Lipids: -C**H$_2$**-CH$_2$-C=O or -C**H$_2$**-CH$_2$-CH=CH- in FAC
**VAR 209**	**1.535 - 1.572**	Lysine<br>Lipids: -C**H$_2$**-CH$_2$-C=O or -C**H$_2$**-CH$_2$-CH=CH- in FAC
**VAR 210**	**1.509 - 1.535**	Isoleucine<br>Lysine
**VAR 211**	**1.489 - 1.509**	Alanine<br>Isoleucine<br>Lysine
**VAR 212**	**1.446 - 1.489**	3-methyl-2-oxopentanoate<br>Isoleucine<br>Lysine
**VAR 213**	**1.408 - 1.446**	3-methyl-2-oxopentanoate<br>Lysine<br>NI 19
**VAR 214**	**1.358 - 1.405**	Lipids: CH$_3$-(C**H$_2$**)$_n$- in FAC

**VAR 215**	**1.338 - 1.358**	Lactate<br>Threonine<br>Lipids: CH_3-(C**H_2**)$_n$- in FAC
**VAR 216**	**1.296 - 1.338**	Lipids: CH_3-(C**H_2**)$_n$- in FAC
**VAR 217**	**1.289 - 1.296**	Isoleucine<br>Lipids: CH_3-(C**H_2**)$_n$- in FAC
**VAR 218**	**1.285 - 1.289**	3-hydroxy-3-methylbutyrate<br>Isoleucine<br>Lipids: CH_3-(C**H_2**)$_n$- in FAC
**VAR 219**	**1.226 - 1.285**	Isoleucine<br>Lipids: CH_3-(C**H_2**)$_n$- in FAC
**VAR 220**	**1.211 - 1.226**	Isoleucine<br>β-Hydroxybutyrate<br>Lipids: CH_3-(C**H_2**)$_n$- in FAC
**VAR 221**	**1.200 - 1.211**	Lipids: CH_3-(C**H_2**)$_n$- in FAC
**VAR 222**	**1.182 - 1.198**	Isopropanol
**VAR 223**	**1.128 - 1.151**	3-methyl-2-oxobutyrate
**VAR 224**	**1.103 - 1.123**	3-methyl-2-oxopentanoate
**VAR 225**	**1.078 - 1.101**	NI 20
**VAR 226**	**1.052 - 1.072**	Valine
**VAR 227**	**1.020 - 1.039**	Isoleucine
**VAR 228**	**1.008 - 1.020**	Valine<br>2-aminobutyrate
**VAR 229**	**1.001 - 1.008**	Valine
**VAR 230**	**0.996 - 1.001**	2-aminobutyrate
**VAR 231**	**0.984 - 0.996**	2-hydroxy-3-methylbutyrate<br>Leucine<br>2-aminobutyrate
**VAR 232**	**0.976 - 0.984**	2-hydroxy-3-methylbutyrate<br>Leucine
**VAR 233**	**0.964 - 0.976**	Isoleucine<br>Leucine
**VAR 234**	**0.938 - 0.964**	4-methyl-2-oxovalerate<br>Isoleucine
**VAR 235**	**0.929 - 0.938**	2-hydroxybutyrate
**VAR 236**	**0.882 - 0.929**	2-hydroxybutyrate<br>3-methyl-2-oxopentanoate<br>Lipids: C**H_3**-(CH_2)$_n$- in FAC
**VAR 237**	**0.800 - 0.882**	2-hydroxy-3-methylbutyrate<br>Lipids: C**H_3**-(CH_2)$_n$- in FAC
